# Microarray profiling shows distinct differences between primary tumors and commonly used preclinical models in hepatocellular carcinoma

**DOI:** 10.1186/s12885-015-1814-8

**Published:** 2015-10-31

**Authors:** Weining Wang, N. Gopalakrishna Iyer, Hsien Ts’ung Tay, Yonghui Wu, Tony K. H. Lim, Lin Zheng, In Chin Song, Chee Keong Kwoh, Hung Huynh, Patrick O. B. Tan, Pierce K. H. Chow

**Affiliations:** 1 Cellular and Molecular Research, National Cancer Centre, Singapore, 169610 Singapore; 2 Department of Surgical Oncology, National Cancer Centre Singapore, Singapore, 169610 Singapore; 3 Department of General Surgery, Singapore General Hospital, 11 Hospital Drive, Singapore, 169608 Singapore; 4 Department of Histopathology, Singapore General Hospital, 11 Hospital Drive, Singapore, 169608 Singapore; 5 SingHealth Experimental Medicine Centre (SEMC), Blk 9, Level 3, Outram Road, Singapore, 169608 Singapore; 6 Division of Information Systems, School of Computer Engineering, Nanyang Technological University, Nanyang Avenue, Singapore, 639798 Singapore; 7 Laboratory of Molecular Endocrinology, Division of Molecular and Cellular Research, National Cancer Centre, 11 Hospital Drive, Singapore, 169610 Singapore; 8 Cancer and Stem Cell Biology Program, Duke-NUS Graduate Medical School, 8 College Road, Singapore, 169857 Singapore; 9 Program in Translational and Clinical Liver Research, National Cancer Centre Singapore, Singapore, 169610 Singapore; 10 Office of Clinical Sciences, Duke-NUS Graduate Medical School, 8 College Road, Singapore, 169857 Singapore

**Keywords:** Hepatocellular carcinoma, Ectopic, Orthotopic, Xenograft, HepG2 cell line

## Abstract

**Background:**

Despite advances in therapeutics, outcomes for hepatocellular carcinoma (HCC) remain poor and there is an urgent need for efficacious systemic therapy. Unfortunately, drugs that are successful in preclinical studies often fail in the clinical setting, and we hypothesize that this is due to functional differences between primary tumors and commonly used preclinical models. In this study, we attempt to answer this question by comparing tumor morphology and gene expression profiles between primary tumors, xenografts and HCC cell lines.

**Methods:**

Hep G2 cell lines and tumor cells from patient tumor explants were subcutaneously (ectopically) injected into the flank and orthotopically into liver parenchyma of Mus Musculus SCID mice. The mice were euthanized after two weeks. RNA was extracted from the tumors, and gene expression profiling was performed using the Gene Chip Human Genome U133 Plus 2.0. Principal component analyses (PCA) and construction of dendrograms were conducted using Partek genomics suite.

**Results:**

PCA showed that the commonly used HepG2 cell line model and its xenograft counterparts were vastly different from all fresh primary tumors. Expression profiles of primary tumors were also significantly divergent from their counterpart patient-derived xenograft (PDX) models, regardless of the site of implantation. Xenografts from the same primary tumors were more likely to cluster together regardless of site of implantation, although heat maps showed distinct differences in gene expression profiles between orthotopic and ectopic models.

**Conclusions:**

The data presented here challenges the utility of routinely used preclinical models. Models using HepG2 were vastly different from primary tumors and PDXs, suggesting that this is not clinically representative. Surprisingly, site of implantation (orthotopic versus ectopic) resulted in limited impact on gene expression profiles, and in both scenarios xenografts differed significantly from the original primary tumors, challenging the long-held notion that orthotopic PDX model is the gold standard preclinical model for HCC.

**Electronic supplementary material:**

The online version of this article (doi:10.1186/s12885-015-1814-8) contains supplementary material, which is available to authorized users.

## Background

HCC is the third most common cause of cancer-related death [1–3], and the sixth most common cancer worldwide owing to increases in the prevalence of hepatitis B virus (HBV) and hepatitis C virus (HCV) [4–10]. The incidence is alarmingly high in the developing world and is rising steadily across the developed world [11, 12]. Three quarters of all HCC occurs in Asian countries due to high local prevalence of chronic HBV infection [13–15]. High incidence regions include sub-Saharan Africa, East Asia and Southeast Asia (Singapore, China, Hong Kong, Taiwan, Korea and Japan) [16–18]. In general, HCC poses a great health threat in the Asia Pacific region [19, 20]. Surgery provides the best cure for HCC, without which demise usually occurs within 6 to 9 months [21]. Even after liver resection, overall prognosis is poor [22–24]. 1-year survival rates after surgical resection are 80-90 %, falling to 41-74 % at 5 years [12, 25, 26]. Unfortunately less than 20 % of patients are surgical candidates because of advanced disease stage at presentation. Even with surgery, up to 80 % of patients develop recurrence within five years of resection [24, 27–29].

Advanced HCC is refractory to conventional chemotherapies and the current standard-of-care sorafenib confers a human survival advantage of only 2.8 months despite tumor regression and suppression of metastasis in mice [30, 31]. Hence, there is an urgent need for an efficacious systemic therapy in both the palliative and adjuvant settings. Advances in understanding of the pathophysiological and molecular basis of HCC have been unmatched by major pharmacological success. Encouraging preclinical animal results all too often fail to translate into human success, and only 45 % of clinical agents showing xenograft responses exhibit clinical activity [32].

Unfortunately, encouraging results from preclinical trials do not often translate into similar successes in patients. We postulate that this disparity could be due to the use of transformed immortalized commercial cell line xenografts and the ectopic implantation of animal models. In contrast, commercial cell lines have a strong track record in cancer research. They are easy to use, readily available and produce reproducible results. However, not all cancers can be immortalized. Those that do accumulate mutations with increasing passages as they adapt to the artificial environment they are cultivated in. Consequently, they often differ genetically and phenotypically from their originating tumors [33]. There is also the well-documented risk of cross-contamination of cell cultures from cells of different origins. Similarly, the subcutaneous implantation in murine models is also favoured in preclinical studies because it is easy to establish and manage and lends itself readily to quantization of tumor burden [34, 35]. However, *in vivo* anti-tumor activity in preclinical animal models does not correlate closely with therapeutic response in human cancers of the same histology [32]. Many authors have also highlighted the importance of tumor microenvironment on the biological behaviour of tumor cells [36]. Despite being more time-consuming, expensive and technically expensive, it has been suggested that orthotopic transplantation of tumor cells into the anatomical site where the cancer commonly arises will give a model which mimics the biological behaviour of tumor cells more closely. This is especially important as the interactions between host environment and tumor graft determine tumor cell expression profiles, levels of growth factors and nutrients, angiogenesis and metastasis [37].

In this study, we investigate and compare the effects of two factors (cell lines vs. patient explants; ectopic vs. orthotopic) on gene expression profiles of HCC tumors. We hypothesized that differences would be observed in gene expression between ectopically and orthotopically transplanted tumors, and also between fresh patient-explanted tumors and established commercial cell lines. The similarities or differences between the sites and tumor types could potentially direct areas for future study.

## Methods

### Establishment of murine models

This study received ethics board approval from the SingHealth Centralized Institutional Review Board, SingHealth Institutional Animal Care and Use Committee (IACUC) and SingHealth Institutional Biosafety Committee. All mice were maintained according to the Guide for the Care and Use of Laboratory Animals published by the National Institutes of Health, USA.

Hep G2 is an established cell line often used in research pertaining to HCC and was selected for comparison against patient tumor explants in our study [38]. The immortalized cell lines were provided by the National University of Singapore (NUS) Biochemistry Laboratory. These had been previously passaged through several generations after purchase from ATCC. Tumor tissue was obtained intraoperatively during liver resection from three patients with prior written informed consent from Department of General Surgery, Singapore General Hospital. All three patients had hepatocellular carcinoma confirmed by histology. The explants were named explant 261004, 21318 and 01–0207. Sections weighing approximately 300 mg were minced into 1–2 mm^3^ fragments using surgical blades, filtered through an 18 Gauge needle and washed 3 times with RPMI1640 before suspension in 0.1 ml of RPMI medium. These cells were then passaged through 5 generations before ectopic or orthotopic implantation into 9 pairs of mice.

Mus Musculus SCID mice were purchased from Animal Resources Centre, Australia. They were maintained for 8 to 10 weeks before experiments in facilities approved by IACUC and the approving ethical committee was SingHealth IACUC. No animal research was conducted outside of the country of residence.

The mice were divided into four groups – ectopic models with Hep G2 cells or cells derived from tumor explants and orthotropic models with Hep G2 cells or cells derived from tumor explants. In order to establish ectopic models, cells were injected subcutaneously into the flank regions of 4 pairs of mice. For orthotopic models, 8 pairs of mice were anaesthetized pre-operatively using intraperitoneal injection of 50 mg/kg/5 mg/kg of ketamine/diazepam solution followed by intramuscular injection of 5 mg/kg of baytril. After anesthetization, the left lobe of liver was exposed through midline abdominal incision and cells were injected directly into liver parenchyma of 4 pairs of mice for each orthotopic model. At any given time during the research study, animals suffering severe or chronic pain or unrelievable distress were painlessly euthanized.

In subcutaneous models, tumor size was measured using callipers. On the other hand, the tumor size of the mice in orthotopic models was monitored and imaged using an R4 microPET scanner (Concordes Microsystems Inc., Knoxville, Tenn., USA). Prior to scanning, the mouse was anaesthetized using 2 % isoflurane. 150–200 uCi of 18F-fluorodeoxyglucose ([18F]-FDG) was injected via the tail vein and conscious uptake was allowed for an hour prior to scanning. The mouse was placed in prone position and static scanning was done for 10 min. Analysis and quantitation of the microPET images were done using Asipro software.

At 2 weeks post-inoculation, tumor size had increased to approximately 1–2 cm in both models. The mice were then euthanized and the tumors were harvested.

### RNA extraction, assessment of nucleic acid purity

20-30 g of fresh tumor tissue was harvested from each mouse for RNA extraction. RNA was also extracted from primary tumours. RNA samples were extracted and purified from tumors using methods described in the RNeasy Mini-Handbook [39]. To negate the random expression changes that may be specific to a xenograft, tissue samples from randomly paired mice within each subgroup were pooled together. RNA concentration and nucleotide purity were confirmed by measuring absorbance at 260 and 280 nm using the SHIMADZU UV-1700 Pharma Spec spectrophotometer. Samples with QC < 1.8 were excluded from the analysis.

### Microarray analysis

We used an Affymetrix GeneChip platform with single channel technology. Extracted RNA was labelled with streptavidin-phycoerythrin conjugate (SAPE) and hybridized to Affymetrix Gene Chip Human Genome U133 Plus 2.0 single arrays. These arrays were scanned using GeneChip Scanner 3000 and images are produced and analysed to give an intensity level for each probe. The intensity level corresponds to the level of hybridisation that occurs for each probe. The raw CEL files were then background corrected and normalized using the Robust Multichip Array (RMA) algorithm [39, 40]. PCA was performed and one way ANOVA was used to partition and identify the major sources of variation. Major non-biological effects (batch effects) influencing gene expression values were tested and corrected for using “Partek Genomics Suite” software. Unsupervised hierarchical clustering was then performed using average linkage and Euclidean distance between intensity readings of all probes as the distance metric. A dendrogram was constructed to illustrate the data.

To compare the differences in gene expressions between orthotopic and ectopic models within each explant/cell line, one-way ANOVA was performed on all gene probes. A false discovery rate (FDR) of less than 0.1 was considered significant. Heat maps were also generated from the RNA microarray data.

## Results

**Fig. 1:**
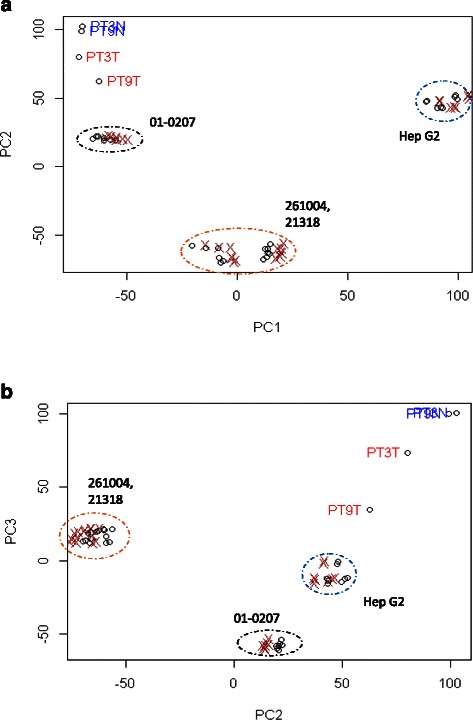
PCA analysis of hierachial clustering of originating tumors, cell line and xenograft samples. **a** PC2 versus PC1, **b** PC3 versus PC2. The originating tumors are represented by spots annotated PTXX. Xenograft and cell lines are segregated from originating tumors. When compared within the murine models, Hep G2 and xenografts from the same primary tumors cluster together regardless of site of implantation

**Fig. 2:**
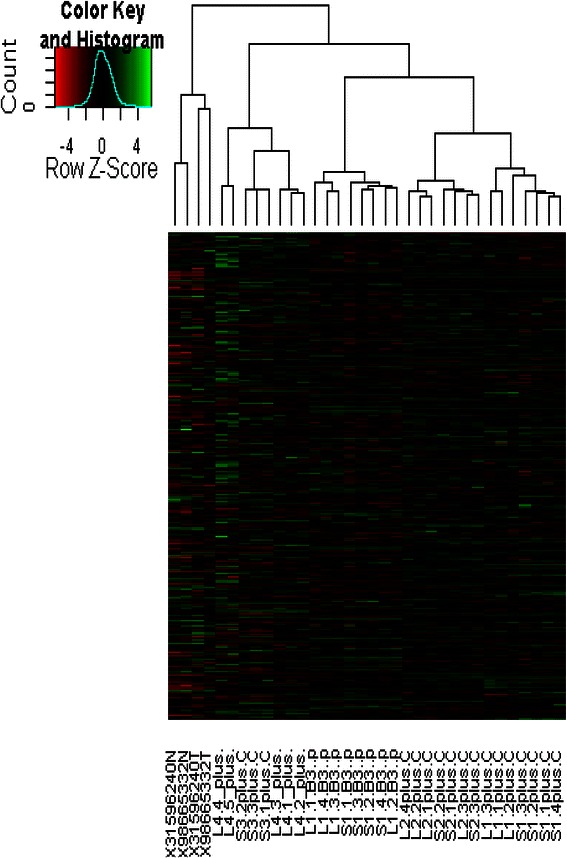
Dendrogram showing clustering of originating tumors, cell line and xenograft samples. There was a distinct separation between originating tumors and samples from murine models but xenografts derived from the same originating tumor clustered together regardless of implantation site. The Hep G2 cell line samples were distinct from patient-derived xenografts

Both the PCA map (Fig. 1) and the dendrogram (Fig. 2) showed the fresh, primary tumors segregating into distinctly separate clusters from all other models, suggesting that primary tumors were genetically distinct from both the immortalised cell line and xenograft models, whether orthotopically or ectopically implanted. When comparing within the murine models, there was a genetic disparity between the HepG2 cell line model and the explant models.

Additionally, the dendrogram showed that the samples of all three patient explant models clustered according to their originating explant, suggesting that tumors were most closely related to other tumors derived from the same originating tumor rather than the site of implantation or the respective primary tumors themselves. However, when comparing within the same tumor explant, there was no difference between orthotopically and ectopically implanted tumors. This corroborates with what is seen on the PCA map.

**Fig. 3:**
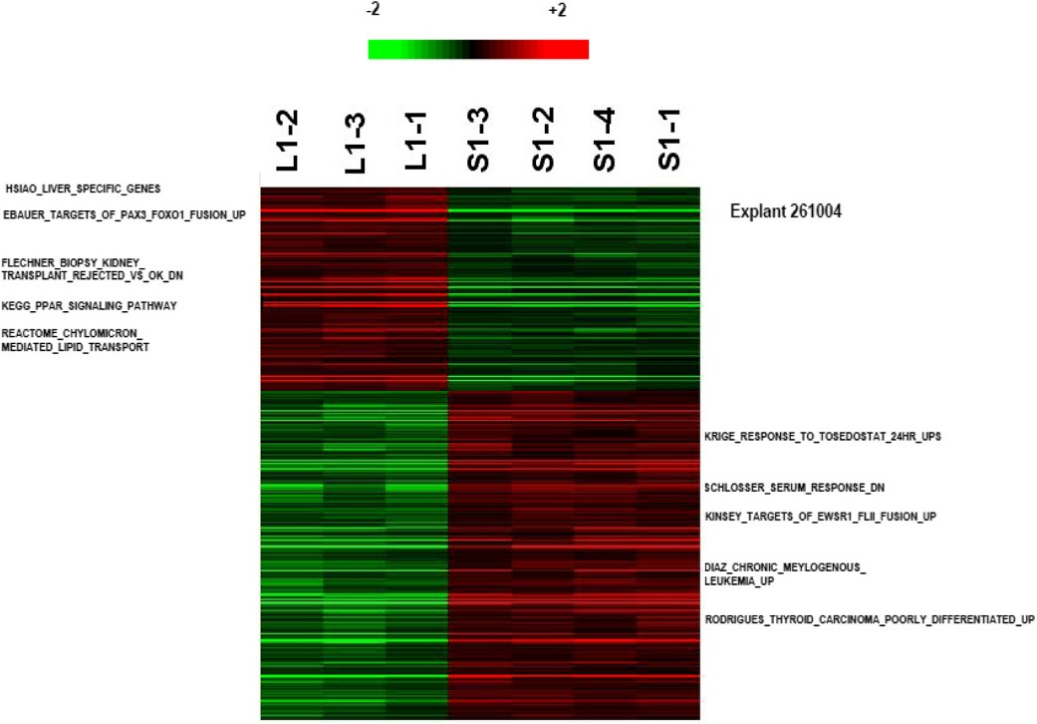
Heat map comparing ectopic and orthotopic models of explant 261004. 396 probes and 56 probes were found to be significantly different between orthotopic and ectopic models of explant 261004

**Fig. 4:**
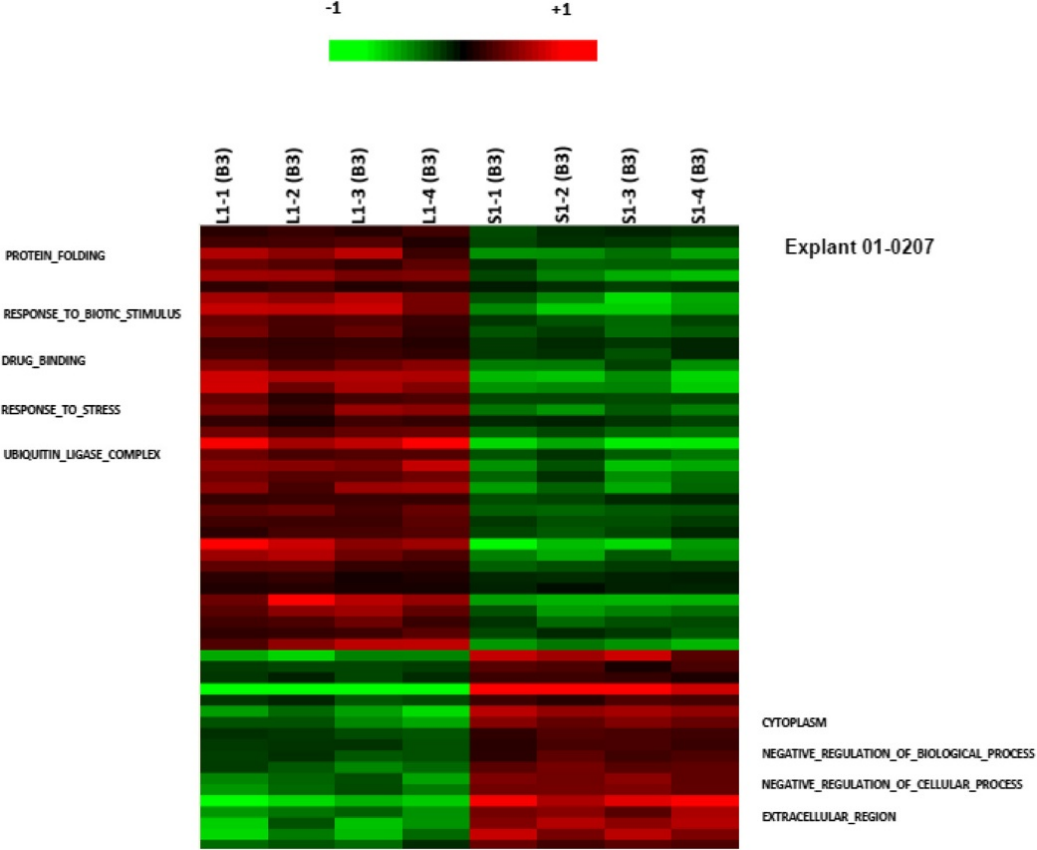
Heat map comparing ectopic and orthotopic models of explant 01–0207. 396 probes and 56 probes were found to be significantly different between orthotopic and ectopic models of explant 01–0207

**Fig. 5:**
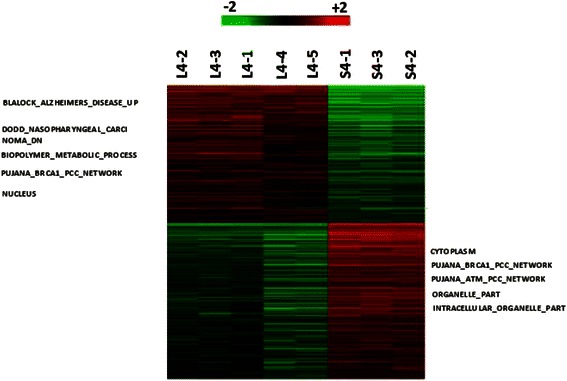
Heat map comparing ectopic and orthotopic models of Hep G2. 17,933 probes were significantly different between both models of Hep G2

One-way ANOVA was performed on all 54,675 gene probes to compare the differences in gene expressions between the orthotopic and ectopic models within each explant/cell line. These results are demonstrated in the heat maps shown (Figs. 3, 4, and 5; Additional file 1). No probes were significantly different between orthotopic and ectopic samples of explant 21318. 396 probes and 56 probes were found to be significantly different for explants 261004 and 01–0207 respectively (Figs. 3 and 4; Additional files 2 and 3). More notably, 17,993 probes were found to be significantly different between orthotopic and ectopic models of Hep G2 (Fig. 5; Additional file 4).

Functional analysis of heat map of expression values using Gene Set Enrichment Analysis (GSEA) for explant 261004 showed upregulation of liver specific genes for orthotopically implanted tumors and downregulation for ectopically implanted tumors. As this was only observed in on sample, the difference could be due to reaction of murine enzymes to the xenograft. To prove this, further experiments involving purification of the samples to remove murine cells would be required.

Differences in gene expressions were also observed in orthotopically and subcutaneously inoculated explant 01–0207. Orthotopically-implanted tumors showed upregulation of genes for protein folding, response to biotic stimuli, enhanced drug-binding, response to stress and ubiquitin ligase complex formation. On the other hand, ectopically-implanted tumors showed up-regulation of genes for negative regulation of cellular processes.

## Discussion

One of the biggest frustrations in drug development for HCC is the frequent failure to reproduce spectacular results from preclinical trials in a clinical setting. This has been attributed to the lack of clinically relevant animal models which can accurately predict the effects of a drug in the clinical setting. Currently, the predominant model consists of inoculating cell line suspension subcutaneously. Cell lines have a strong track record in cancer research due to their availability, rapid growth and ease of use. Ectopic models are also cheaper and less technically challenging than their orthotopic counterparts, thus allowing rapid screening of cytotoxic agents. However, it has been suggested that such models underestimate the influence of organ environment on subsequent tumorigenesis and metastasis. In this study, we aim to study the effects of the type of xenograft (cell line vs. patient tumor explant) and the site of xenograft inoculation (orthotopic vs. ectopic) on gene expression profiles and morphology of tumors.

A few conclusions can be drawn from our study. Firstly, gene expression of fresh tumors differed from the cell line and xenograft models, representing a drift in genotypic expression of the xenografts from originating tumors regardless of the site of implantation. It has been previously demonstrated by other authors that selection pressures in both cell-line and patient-derived xenograft models drive tumour growth which favours a more-aggressive sub-clone [41]. Neither model is a faithful representation of the heterogeneity seen in the original tumour which could explain the differences in gene expression seen in our study. Secondly, while cell line xenografts are distinct in gene expression from those of cells derived from patient tumor explants, there is no significant genotypic difference between xenografts that were orthotopically and ectopically implanted. That the cell line xenograft should differ from those derived from tumor explants is not a surprising conclusion having been previously demonstrated by several groups [42]. This lends credence to hypotheses that cell lines can become markedly unrepresentative of their parent tumors over time. Therefore, while cell lines are popular in research, our results show that they might be poor candidates as clinically relevant models in pharmacological studies. Cell lines are forced to adapt to a vastly different environment and this could lead to important characteristics of the original tumor to be selected against and eventually lost. Furthermore, there is also chance of cross-contamination of cultures and accumulation of mutations through passages [36, 42, 43]. However, we have only tested one cell line in this study and it is important to note that similar results may not hold true in other cell lines. More work still needs to be done to evaluate reproducibility in other established cell lines or discover and exclude those that hamper our efforts to bridge the preclinical-clinical divide.

In our study, the heat maps showed differences in orthotopically and ectopically implanted cell line models and 2 of the 3 patient explant models (261004 and 01–0207). Ectopically-implanted tumors appeared less metabolically active, responded less to biotic stimuli and external stresses, did not bind drugs and expressed higher levels of ubiquitin ligase, an enzyme involved in protein degradation. These could suggest biological bases for observed differences in preclinical and clinical drug response.

However, these differences in gene expression were not significant enough to cause a divergence in the dendrogram and PCA map. Tumors derived from the same originating explant clustered together, suggesting that the site of xenograft implantation has limited impact on gene expression. Other authors have previously shown that drug response was altered between orthotopic and ectopic murine models [44, 45]. Taken together, the data suggests multifactorial influence on pharmacodynamics effects but organ microenvironment and tumor-host interactions could have more profound effects on drug response rather than gene expression. Indeed, studies have shown that invasive genotype and phenotype are affected by organ environment [44, 46]. Tumors transplanted subcutaneously were observed to be well-encapsulated, even when such feature was not apparent in the original tumor [47].

These results have implications on the choice of appropriate models. Although ectopic models have their limitations, our results showed that there is not much difference genetically between orthotopic and ectopic models. Unless organ-specific cellular targets are involved, ectopic models should still be considered for initial screening of drug target for their speed, ease of use and lower cost. Ectopic models would, however, be lacking in experiments studying the metastatic potential of HCC and the effect of autocrine and paracrine growth factors on the tumor. Furthermore, the liver is a complex organ with a vast and unique vasculature. The slow but extensive vasculature provides a favourable environment for tumors to establish, thrive and metastasize [44]. In addition, proper functioning of liver in HCC patients is often complicated by cirrhosis, adding another layer of complexity which cannot be replicated in a subcutaneous model. An orthotopic model places the tumor in its native environment, allowing more evaluation of exposure levels of drug to tumor at an organ level, the rate of growth in the natural milieu and other tumor-host interactions. Therefore, the orthotopic model could be used to validate potential drug targets after initial screening.

## Conclusions

In summary, our study showed that Hep G2 cells are genetically different from the other xenograft models. They also exhibited markedly different gene expression levels between orthotopic and ectopic sites and are probably a poor experimental choice for representing HCC. Tumor samples derived from patient explant clustered together according to their originating tumor. Heat maps showed different gene expression levels in explants 261004 and 01–0207 but these differences did not cause divergence of ectopic and orthotopic models on PCA mapping. This demonstrates that, despite the limitations, there could still be a role for ectopic models in drug screening, especially when its lower cost, rapidity and ease of use are considered. The lack of divergence in gene expression between orthotopic and ectopic models also suggests that tumor microenvironment and host-tumor interactions may have a greater impact on preclinical and clinical drug response disparity than gene expression. We suggest that ectopic models and orthotopic models can be complementary in their use; with ectopic models being used in initial target screening and orthotopic models used in the validation of potential drug targets or when more subtle organ-specific aspects need to be studied.

### Availability of supporting data

The microarray data sets supporting the results of this article are available in the NCBI’s Gene Expression Omnibus repository, http://www.ncbi.nlm.nih.gov/geo/query/acc.cgi?acc=GSE72981. Other data sets supporting the results of this article are included within the article and its additional files published through LabArchives, DOI:10.6070/H4K07297 and hyperlink in https://mynotebook.labarchives.com/share/piercechow/MjAuOHwxMTg1NjMvMTYvVHJlZU5vZGUvMjUwNTk0MTM3MHw1Mi44.

## Additional files

Additional file 1: **Venn diagram illustrating the differences in gene expression in explant 261004, explant 01-0207 and Hep G2.** (DOCX 365 kb)
Additional file 2: **Expression profiling data of explant 261004.** (XLS 110 kb)
Additional file 3: **Expression profiling data of explant 01-0207.** (XLS 38 kb)
Additional file 4: **Expression profiling data of explant HEP G2.** (XLS 2175 kb)

## Abbreviations

HCC: Hepatocellular carcinoma
PCA: Principal component analyses
PDX: Patient-derived xenograft
HBV: Hepatitis B virus
HCV: Hepatitis C virus
SAPE: streptavidin-phycoerythrin conjugate
RMA: Robust Multichip Array
FDR: False discovery rate
GSEA: Gene set enrichment analysis
