# Supplementary figures and images for: Microarray profiling shows distinct differences between primary tumors and commonly used preclinical models in hepatocellular carcinoma
Source: BMC Cancer. 2015 Oct 31;15:828. doi: 10.1186/s12885-015-1814-8 (PMC4628260; doi:10.1186/s12885-015-1814-8)

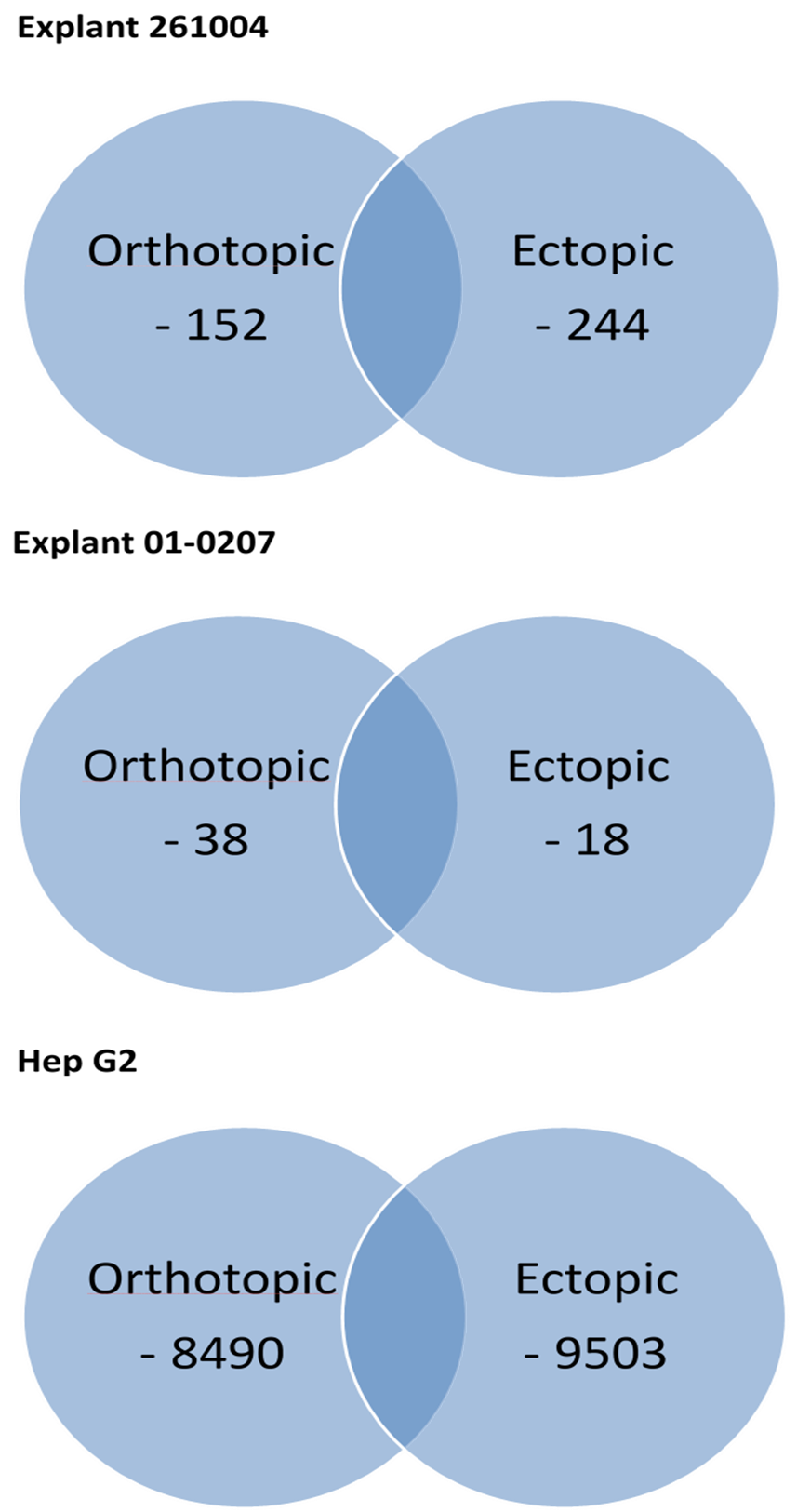

Supplement: Additional file 1: — Venn diagram illustrating the differences in gene expression in explant 261004, explant 01-0207 and Hep G2. (DOCX 365 kb) [file 12885_2015_1814_MOESM1_ESM.docx]
